# Editorial on the Special Issue “Advances in Pediatric Acute Kidney Injury”

**DOI:** 10.3390/children11020195

**Published:** 2024-02-03

**Authors:** Giulio Rivetti, Paolo Montaldo, Pierluigi Marzuillo

**Affiliations:** Department of Woman, Child and of General and Specialized Surgery, Università degli Studi della Campania “Luigi Vanvitelli”, Via Luigi De Crecchio 2, 80138 Naples, Italy; giuliorivetti94@gmail.com (G.R.); p.montaldo@imperial.ac.uk (P.M.)

Acute kidney injury (AKI) refers to a swift decline in kidney function, marked by the reduced excretion of waste products and disturbances in fluid and electrolyte balance [1,2]. The Kidney Disease/Improving Global Outcome (KDIGO) guidelines identify AKI based on elevated serum creatinine levels or decreased urine outputs [2].

Despite its significant impact on long-term kidney health [3,4], AKI often goes unnoticed in pediatric cases [5,6]. The severity of AKI is closely associated with both short- and long-term adverse outcomes [7,8,9,10], with animal studies indicating potential long-term structural changes such as renal fibrosis [11]. This leads to a doubled risk of chronic kidney disease (CKD) even with mild AKI; the risk rises exponentially with AKI severity [3]. However, AKI is commonly observed in hospitalized children, complicating 1–25% of intensive care unit (ICU) admissions and 1–7% of total hospital admissions [12]. This Special Issue, entitled “Advances in pediatric acute kidney injury”, aims to enhance our understanding of AKI in children and neonates. This Editorial focuses on the innovations presented in the articles featured in this Special Issue.

AKI can complicate various common pediatric conditions [13,14,15,16,17,18,19,20]. For example, children with type one diabetes mellitus (T1DM) may undergo kidney damage in both chronic and acute settings [21,22]. Osmotic polyuria from hyperglycemia during T1DM onset can lead to dehydration, hypovolemia, and kidney hypoperfusion, resulting in tubular damage [21].

During T1DM onset, AKI may occur in 43.8% of patients, reaching 65% in those with diabetic ketoacidosis [16]. Acidosis can expedite tubular damage deterioration [21].

The baseline serum creatinine (bSCr) level is critical for AKI diagnosis, and when the measured bSCr (mbSCr) is unavailable, estimated bSCr (ebSCr) based on height can serve as a viable substitute [23]. Depending on the method used to quantify creatinine, the revised Schwartz equation [24] or the original Schwartz equation [25] is employed.

In this context, Guarino et al. assessed whether the ebSCr, calculated based on height, could serve as a viable alternative for diagnosing AKI compared with the mbSCr. This investigation was conducted within a cohort of individuals experiencing onset of T1DM, as presented in this Special Issue [23]. Notably, the study revealed a substantial 90% agreement between ebSCr and the established gold standard, the mbSCr value [23]. This underscores the potential use of ebSCr based on height as a screening tool for identifying AKI in patients experiencing T1DM onset, especially in situations where mbSCr is unavailable, such as in critically ill children in pediatric intensive care units (PICUs). Additionally, the study recommended using the revised Schwartz equation [24] for the retrocalculation of creatinine based on height when the creatinine is determined using the IDMS-traceable technique [eb-SCr (mg/dL) = (0.413 height [cm])/baseline estimated glomerular filtration rate (eGFR)]. However, if the Jaffe technique is employed to quantify creatinine, use of the original Schwartz equation [25] is advised [23].

In a concise report by Marzuillo et al., the prevalence and risk factors associated with AKI in a cohort of patients with acute appendicitis (AA) were explored. Among 122 children hospitalized for AA, AKI occurred in 7.4%, and it was associated with vomiting, 5% dehydration, axillary body temperature ≥ 38.5 °C, and elevated levels of C-reactive protein and neutrophils [13].

As mentioned earlier, AKI doubles the risk of chronic kidney disease [3], and conditions linked to reduced nephronic mass significantly contribute to AKI development [15].

For example, very-low-birth-weight babies are at an increased risk due to insufficient renal reserves, stressful postnatal events, and medication exposures [26]. Lazarovits et al. reported a 21% prevalence of AKI in 152 infants with very low birth weight, with predictors including the use of vasopressors, patent ductus arteriosus, and bloodstream infection [26]. Neonatal mortality and AKI were strongly correlated [26]. Various methods exist to diagnose AKI [9,27], such as the widely used risk, injury, failure, loss, and end-stage (RIFLE) criteria [28]. The “Acute Kidney Injury Network” (AKIN) developed a classification system which evaluates changes in kidney function within 48 h to increase the sensitivity of the RIFLE detection approach [29].

To enhance the sensitivity of RIFLE, the AKIN introduced a categorization system to assess changes in kidney function within a 48 h period [29]. Leblebici et al. conducted a study comparing AKIN with pediatric RIFLE (pRIFLE) AKI classifications and PICU scoring methods. In the original article by Leblebici et al., it was revealed that 8.7% of the PICU patients developed kidney injury according to pRIFLE and AKIN classifications, significantly impacting mortality [29]. Renal injury incidence exhibited a strong statistical correlation with elevated Pediatric Mortality Risk Score (PRISM III), Pediatric Logistic Organ Dysfunction-2 (PELOD-2), and Pediatric Sequential Organ Failure Assessment (pSOFA) scores. When comparing AKIN criteria stages with PRISM III, PELOD-2, and pSOFA scores, a significant difference was observed between patients without AKI and those with stage 1, stage 2, and stage 3 kidney injury. However, for PRISM III, PELOD-2, and pSOFA scores, no significant differences were found between stages, according to AKIN criteria [29]. Notably, a substantial difference emerged between patients without AKI and those in the risk, injury, and failure plus loss stages according to pRIFLE criteria. According to the Pediatric Mortality Index (PIM-2) ratio and pRIFLE criteria, a statistically significant difference was identified between patients in the injury and failure plus loss stages and those without AKI [29].

The association between kidney impairment and COVID-19 infection has also been acknowledged [30,31,32]. In their systematic review, Wu et al. shared findings from a thorough literature investigation on renal complications arising from COVID-19 in children and adolescents. The most commonly reported manifestation was nephrotic syndrome, and there were additional instances involving diverse glomerulonephritides [33]. Furthermore, individuals with transplanted kidneys who tested positive for COVID-19 displayed T-cell-mediated rejection and moderate tubular interstitial infiltration [33].

Finally, in a separate comprehensive investigation, Wu et al. documented the potential negative impacts of COVID-19 vaccination on the kidneys within a restricted group of vaccinated children and adolescents, a subset of the wider vaccinated population. They emphasized that, unless specific circumstances dictate otherwise, there is currently no justification to abstain from vaccination, given that the advantages far exceed any potential risks [34]. The authors particularly advocated for the careful monitoring of all children and adolescents undergoing COVID-19 vaccination [34].

We hope readers are satisfied with the new evidence in this area of research.
